# Supplementary material for: Benchmarking large language models for predictive modeling in biomedical research with a focus on reproductive health
Source: Cell Rep Med. 2026 Feb 17;7(2):102594. doi: 10.1016/j.xcrm.2026.102594 (PMC12923944; doi:10.1016/j.xcrm.2026.102594)
Supplement: Document S1. Tables S1–S5 [file mmc1.pdf]

Cell Reports Medicine, Volume *7*

Supplemental information

Benchmarking large language models

for predictive modeling in biomedical research

with a focus on reproductive health

Reuben Sarwal, Victor Tarca, Claire A. Dubin, Nikolas Kalavros, Gaurav Bhatti, Sanchita Bhattacharya, Atul Butte, Roberto Romero, Gustavo Stolovitzky, Tomiko T. Oskotsky, Adi L. Tarca, and Marina Sirota

Supplementary Tables

Table S1: LLMs used in the comparison

LLM	Developer	Parameters	Run Method
Deepseek-R1	DeepSeek	671B	API
4o	OpenAI	Unknown	API
o3-mini-high	OpenAI	Unknown	API
Gemini2.0 FlashExpThink	Google	Unknown	API
Qwen2.5 Coder	Alibaba	14B	Local
Llama3.2	Meta	3B	Local
Phi-4	Microsoft	14B	Local
DeepSeek-R1-Distill-Qwen	DeepSeek	32B	Local

Table S1: Details the eight different LLMs tested for generating code for the 8 different prediction tasks. Developers of each LLM are listed, as well as the method by which the LLM was run (API vs. Local). When available, information on the number of parameters for each LLM is described as well. While GPT-4o is OpenAI's flagship multimodal model, designed for both high-level reasoning and seamless real-time multimodal interaction, o3-mini-high is a more specialized model for technical/logical reasoning within text—best used for accuracy on STEM, math, and programming. Sources: https://openai.com/index/openai-o3-mini/, https://openai.com/index/hello-gpt-4o/. **Related to** Figure 1 and STAR methods.

Table S2: Prompts and computational tasks

Task/Data type	Prompt to LLM
Q1:<br>Predict gestational age from gene expression in blood<br><br>~30k features	Write a program in **R/python** which downloads the Gene Expression Omnibus dataset GSE149440 and uses expression data of tens of thousands of genes in a few hundred samples to fit a prediction model of gestational age (gestational age:ch1) variable from the metadata. Only samples assigned to the training set (metadata variable train:ch1=='1') should be used for training. Apply the model on the test defined as metadata variable train:ch1==0. Print the root mean squared error (RMSE) on the test set and generate a scatter plot of the predicted vs actual gestational age values for the test set.
Q2<br><br>Predict gestational age from placenta methylation data<br><br>~360k features	Write **R/python** code that fits one predictive model for a continuous variable gestational age (GA) using about 400 thousand methylation features measured in about 2000 samples. The feature data for the training set is in df_training.csv while for the test set is in df_test.csv. Features are rows and samples are columns in the feature data, with the first column being the feature names. The metadata files are ano_training.csv and ano_test.csv. The feature data and metadata are linked by the Sample_ID. Do not use any other metadata variables in the models besides feature data. Print the root mean squared error (RMSE) on the test set and generate a scatter plot of the predicted vs actual gestational age values for the test set and include RMSE in the legend.
Q3A:<br>Predict Preterm Birth from microbial abundance<br><br>Q3B:<br>Predict Early Preterm Birth from microbial abundance<br><br>~2k features	Write **R/python** code that fits one predictive model for a binary outcome PTB using about two thousands microbiome features measured in a few hundreds of samples. The feature data for the training set is in training_species_abundance.csv while for the test set is in validation_species_abundance.csv. Discard features not in common between training and validation sets and do not use any other metadata in the models besides microbial feature data. The metadata for the training set is in training_metadata.csv while for the test set in validation_metadata.csv. The feature data and metadata are linked by the specimen column in these datasets. There are multiple observations (specimen) for each subject identified by the column participant_id. Do use only the entry with highest collect_wk value < 32 for each participant_id to fit and evaluate the models. PTB is defined as metadata variable delivery_wk < 37. Print AUC ROC value on the test set and plot an ROC curve. Repeat the analysis above to predict EarlyPTB outcome defined as delivery_wk < 32 using data for last specimen with collect_wk<28 for each participant_id.

Table S2: For each task, the main goal/endpoint is noted, along with the number of features in the three different datasets used. The exact prompt given to the LLMs is shown. **Related to** Figure 1 and STAR methods.

Table S3: Dataset information

Dataset	DREAM Challenge name	Accession Number	Number of samples		
			Train	Test	Total
**Q1**	Predict gestational age from blood transcriptomics data	Syn1838082<br><br>GSE149440	367	368	735
**Q2**	Predict gestational age from placental methylation data	Syn59520082	1742	384	2126
**Q3 (A and B)**	Classify term vs. preterm birth from vaginal microbiome data	Syn26133770<br><br>SDY2187<br><br>SDY465	1895	302	2197

Table S3: This table details the four different tasks each LLM was prompted to perform. Q1 task prompted LLMs to build a model to predict gestational age from transcriptomics data, Q2 prompted LLMs to predict gestational age again, but from methylation data, and Q3 prompted LLMs to build a model to classify preterm birth from microbiome data. The table also details the study accession numbers for each of the datasets, as well as the number of samples in the training, test, and full datasets. Syn is a synapse identifier (synapse.org); GSE is a Gene Expression Omnibus identifier. SDY is an ImmPort identifier (https://immport.org/). **Related to** Figure 1 and STAR Methods.

Table S4: Performance metrics for LLMs and top human models

**Dataset**	**Predictor**	**RMSE/AUC**	**95% Confidence Interval**
Q1	Human	**4.55**	(4.18, 4.96)
Q1	o3-high: R	5.42	(5.04, 5.82)
Q1	4o: R	5.43	(5.06, 5.80)
Q1	DeepseekR1: R	5.43	(5.06, 5.80)
Q2	4o: Python	**1.12**	(1.04, 1.21)
Q2	Human	1.24	(1.16, 1.33)
Q2	o3-high: R	1.47	(1.38, 1.57)
Q2	4o: R	1.52	(1.42, 1.62)
Q2	DeepseekR1: R	1.52	(1.43, 1.61)
Q2	o3-high: Python	1.94	(1.83, 2.05)
Q2	DeepseekR1: Python	1.21	(1.13, 1.29)
Q3A	Human	0.68	(0.58,0.77)
Q3A	4o: R	0.57	(0.47,0.67)
Q3A	Gemini: R	0.57	(0.47,0.67)
Q3A	o3-high: R	0.57	(0.46,0.67)

Q3A	o3-high: Python	0.55	(0.45,0.65)
Q3A	DeepseekR1: R	0.5	(0.40,0.61)
Q3A	Gemini: Python	0.39	(0.29,0.49)
Q3B	Human	**0.92**	(0.86,0.98)
Q3B	4o: R	0.59	(0.34,0.82)
Q3B	Gemini: R	0.59	(0.34,0.81)
Q3B	o3-high: R	0.59	(0.35,0.81)
Q3B	DeepseekR1: R	0.54	(0.33,0.75)
Q3B	Gemini: Python	0.56	(0.34,0.78)
Q3B	o3-high: Python	0.54	(0.39,0.70)

Table S4: Performance metrics with 95% confidence intervals for LMM models and top human models. Values in bold mean significantly better (more accurate) than the next ranked model. **Related to** Figure 2.

39 **Table S5: Reproducibility of o3-mini-high LLM code generation**
40

Task	Language	LLM run	Completed	Metric type	Value
1	R	Primary	1	RMSE	**5.42**
1	R	Repeat 1	1	RMSE	5.43
1	R	Repeat 2	1	RMSE	5.42
1	R	Repeat 3	1	RMSE	5.43
1	Python	Primary	0	RMSE	NA
1	Python	Repeat 1	0	RMSE	NA
1	Python	Repeat 2	0	RMSE	NA
1	Python	Repeat 3	0	RMSE	NA
2	R	Primary	1	RMSE	1.47*
2	R	Repeat 1	1	RMSE	1.51
2	R	Repeat 2	1	RMSE	1.51
2	R	Repeat 3	1	RMSE	1.26
2	Python	Primary	1	RMSE	**1.94**
2	Python	Repeat 1	1	RMSE	1.21
2	Python	Repeat 2	1	RMSE	2.52
2	Python	Repeat 3	1	RMSE	1.94

3A	R	Primary	1	AUC	**0.567**
3A	R	Repeat 1	1	AUC	0.50
3A	R	Repeat 2	1	AUC	0.56
3A	R	Repeat 3	1	AUC	0.56
3A	Python	Primary	1	AUC	0.55**
3A	Python	Repeat 1	1	AUC	0.394
3A	Python	Repeat 2	1	AUC	0.394
3A	Python	Repeat 3	1	AUC	0.394
3B	R	Primary	1	AUC	**0.587**
3B	R	Repeat 1	1	AUC	0.54
3B	R	Repeat 2	1	AUC	0.60
3B	R	Repeat 3	1	AUC	0.60
3B	Python	Primary	1	AUC	**0.54**
3B	Python	Repeat 1	1	AUC	0.54
3B	Python	Repeat 2	1	AUC	0.54
3B	Python	Repeat 3	1	AUC	0.54

Table S5: The completion of the analysis task (1: success; 0: failure) and performance metrics are shown for the primary analysis reported and for 3 additional repeats of analysis code generation. Bolded values of the primary analysis match the average of the 3 additional repeats. * indicates that RMSE in primary analysis was higher (lower accuracy) than the average of the 3 additional repeats. ** indicates that the

AUC value in the primary analysis was higher (better accuracy) than the average of the 3 repeats. **Related to** Figure 2.
