# Supplementary material for: Benchmarking large language models for predictive modeling in biomedical research with a focus on reproductive health
Source: Cell Rep Med. 2026 Feb 17;7(2):102594. doi: 10.1016/j.xcrm.2026.102594 (PMC12923944; doi:10.1016/j.xcrm.2026.102594)
Supplement: Document S2. Article plus supplemental information [file mmc2.pdf]

Cell Reports Medicine

Report

Benchmarking large language models for predictive modeling in biomedical research with a focus on reproductive health

Graphical abstract

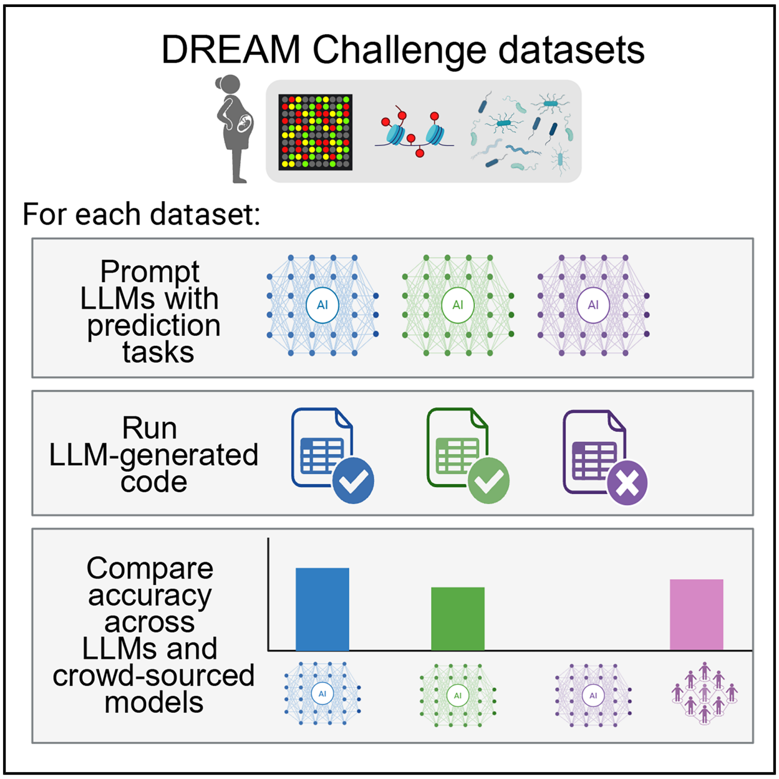

Highlights

- Large language models (LLMs) generate R and Python code for omics prediction tasks
- Top LLM matched or exceeded median DREAM challenge participant performance
- LLMs allowed model development in minutes starting with tabular data
- Single-shot prompting reveals strengths and limitations of LLM-based code generation

Authors

Reuben Sarwal, Victor Tarca, Claire A. Dubin, ..., Tomiko T. Oskotsky, Adi L. Tarca, Marina Sirota

Correspondence

marina.sirota@ucsf.edu

In brief

Sarwal et al. benchmark large language models (LLMs) on four reproductive health prediction tasks from DREAM challenges. Several LLM-generated executable workflows match median participant accuracy and, in one case, outperform top teams. This demonstrates that LLMs can successfully assist with developing predictive machine learning models, while still requiring human oversight.

Sarwal et al., 2026, Cell Reports Medicine 7, 102594
February 17, 2026 © 2026 The Author(s). Published by Elsevier Inc.
https://doi.org/10.1016/j.xcrm.2026.102594

CellPress

Cell Reports Medicine

CellPress
OPEN ACCESS

Report

Benchmarking large language models for predictive modeling in biomedical research with a focus on reproductive health

Reuben Sarwal,[1,15] Victor Tarca,[2,15] Claire A. Dubin,[1] Nikolas Kalavros,[3,4] Gaurav Bhatti,[5] Sanchita Bhattacharya,[1] Atul Butte,[1,14] Roberto Romero,[6,7,8] Gustavo Stolovitzky,[9,10] Tomiko T. Oskotsky,[1,11,16] Adi L. Tarca,[5,12,13,16] and Marina Sirota[1,14,16,17,*]

[1]Bakar Computational Health Sciences Institute, University of California, San Francisco, San Francisco, CA 94158, USA
[2]Huron High School, Ann Arbor, MI 48105, USA
[3]New York University Langone Health, New York, NY 10016, USA
[4]Division of Precision Medicine, Department of Medicine, NYU Grossman School of Medicine, New York, NY 10016, USA
[5]Center for Molecular Medicine and Genetics, Wayne State University School of Medicine, Detroit, MI 48201, USA
[6]Pregnancy Research Branch, Division of Obstetrics and Maternal-Fetal Medicine, Division of Intramural Research, Eunice Kennedy Shriver National Institute of Child Health and Human Development, National Institutes of Health, United States Department of Health and Human Services, Bethesda, MD 20892, USA
[7]Department of Obstetrics and Gynecology, University of Michigan, Ann Arbor, MI 48109, USA
[8]Department of Epidemiology and Biostatistics, Michigan State University, East Lansing, MI 48824, USA
[9]Department of Pathology, New York University Grossman School of Medicine, New York, NY 10016, USA
[10]Biomedical Data Science Hub, New York University Langone Health, New York, NY 10016, USA
[11]Division of Clinical Informatics and Digital Transformation, Department of Medicine, University of California, San Francisco, San Francisco, CA 94143, USA
[12]Department of Obstetrics and Gynecology, Wayne State University School of Medicine, Detroit, MI 48201, USA
[13]Department of Computer Science, Wayne State University College of Engineering, Detroit, MI 48201, USA
[14]Department of Pediatrics, University of California, San Francisco, San Francisco, CA 94143, USA
[15]These authors contributed equally
[16]Senior author
[17]Lead contact
*Correspondence: marina.sirota@ucsf.edu
https://doi.org/10.1016/j.xcrm.2026.102594

SUMMARY

Large language models (LLMs) are increasingly used for code generation and data analysis. This study assesses LLM performance across four predictive tasks from three DREAM challenges: gestational age regression from transcriptomics and DNA methylation and classification of preterm birth and early preterm birth from microbiome data. We prompt LLMs with task descriptions, data locations, and target outcomes and then run LLM-generated code to fit prediction models and determine accuracy on test sets. Among the eight LLMs tested, o3-mini-high, 4o, DeepseekR1, and Gemini 2.0 can complete at least one task. R code generation is more successful (14/16) than Python (7/16). OpenAI's o3-mini-high outperforms others, completing 7/8 tasks. Test set performance of the top LLM-generated models matches or exceeds the median-participating team for all four tasks and surpasses the top-performing team for one task ($p = 0.02$). These findings underscore the potential of LLMs to democratize predictive modeling in omics and increase research output.

INTRODUCTION

Reproductive health is a critical area of study that encompasses fertility, pregnancy, and childbirth, all of which have profound implications for individual and public health. Among these concerns, preterm birth—defined as delivery before 37 weeks of gestation—remains a major global challenge, affecting approximately 11% of infants worldwide and leading to significant short- and long-term health consequences.[1] Understanding and addressing issues in reproductive health is essential for developing effective prevention strategies and promoting maternal and infant well-being. In recent years, predictive models in reproductive medicine have been developed to estimate the likelihood of adverse pregnancy outcomes, supporting personalized care and informed decision-making.[2] However, the performance of these models depends heavily on the quality and size of the datasets used, the distribution and types of data available, and the specific methods applied.[3] Continued research in reproductive health, supported by robust data and innovative technology, is key to improving outcomes across the reproductive lifespan.

Recent advances in maternal health research underscore the critical importance of accurately estimating gestational age[4]

© 2026 The Author(s). Published by Elsevier Inc.
This is an open access article under the CC BY license (http://creativecommons.org/licenses/by/4.0/).

and understanding the molecular mechanisms underlying placental aging.[5] However, current clinical tools such as fetal ultrasound remain imprecise, often estimating gestational age with errors of several weeks[6,7] and only 55.1% delivering within 1 week of their due date.[8] Moreover, there are no reliable predictive tools in practice to assess the risk for preterm delivery or effective treatments to prevent it, besides vaginal progesterone for the subset of women with a short cervix.[9–11] Accurate assessment of gestational age and preterm birth risk would enable better evaluation of fetal maturity and targeted interventions to reduce prematurity risk. Precise determination of gestational age is essential for optimizing clinical management, guiding timely interventions, and reducing adverse outcomes for both mother and child. Equally, insights into the "placental clock"—the epigenetic and molecular processes that regulate placental aging—provide invaluable clues to fetal development, maternal adaptation, and long-term health trajectories.[12] Crowdsourced open science initiatives, exemplified by challenges such as the DREAM (Dialogue for Reverse Engineering Assessments and Methods) challenges,[13] harness global collaboration and diverse datasets, including transcriptomic,[14] microbiome,[15] and methylation data[16] to develop predictive models addressing these critical questions. In particular, DREAM challenges have framed tasks directly aimed at these clinical gaps, using blood transcriptomics to predict gestational age, placental methylation data to model placental aging, and vaginal microbiome profiles to forecast preterm birth risk, highlighting the translational potential of data-driven discovery. While such collaborative approaches offer access to a broad spectrum of data, computational methodologies, and collective expertise, they also face challenges related to coding variability and inconsistent model performance.

Large language models (LLMs) are a promising solution to issues of inconsistency. Cutting-edge systems such as GPT-4, DeepSeek, and others have evolved rapidly, and their applications find critical utility in biological and medical sciences where the challenge of "too much data, too few experts" has long hindered progress.[17,18] By utilizing vast corpora of text-based data—spanning scientific literature, experimental results, and patient records—LLMs are enabling breakthroughs in cancer diagnosis, radiology, electronic health record analysis, hypothesis generation, and more (PathChat,[19] Flamingo-CXR,[20] Med-PaLM[21]). Another application of LLMs is to democratize data analysis to non-expert programmers, streamline analysis, and accelerate the discovery and interpretation process in biomedical data sciences. This has been followed by the widespread adoption of LLMs in all parts of the scientific process. Alongside this exciting explosion in LLM usage comes the need for careful and meticulous evaluation, and researchers have highlighted both their potential for breakthrough discoveries and their potential pitfalls.[22]

Herein, we utilized the wealth of crowdsourced benchmark data from DREAM challenges to assess the ability of LLMs to support the development of code for building predictive models with omics data, and then assess and visualize model performance. Each DREAM challenge consisted of a research question coupled with training and test data. Teams from around the world submitted solutions to tackle a problem based on training data, which were then evaluated on a blinded test set, mirroring the train-test process in machine learning. We prompted various popular LLMs to mirror a typical workflow of a bioinformatician who writes R or Python code to read data from local files or web-based repositories, to build and evaluate a prediction model for multiple tasks. The data types considered included transcriptomic, epigenetic, and microbiome, spanning several orders of magnitude in terms of problem dimensionality. We systematically assessed the LLMs' performance in a single-shot setting using 4 prediction tasks: gestational age prediction from blood gene expression or placenta methylation data and preterm or early preterm birth classification from microbiome data. Executing and evaluating the code generated by LLMs, we derived insight in terms of coding language-specific reliability of the LLM-generated code and their ability to match the test set prediction accuracy of participants in the original DREAM challenges. Our study reframes the usage of LLMs in the context of crowdsourced research efforts, highlighting their ability to generate reproducible machine learning pipelines that reduce inconsistencies and improve efficiency in a multi-team collaborative setting.

RESULTS

Study overview

The methodology used in this study is depicted in Figure 1. Briefly, each of the 8 LLMs considered (Table S1) was prompted to generate R or Python code that uses one training dataset that (1) fits a model for the given task, (2) applies the model to the corresponding test set to calculate an appropriate performance metric, and (3) generates a visualization of the result (Table S2). These prompts specified the type and source of data, dimensionality of the feature space, prediction outcome, data partitioning into training and test sets, and required model evaluation metrics (root-mean-square error [RMSE] for continuous outcomes and area under the receiver operating characteristic curve [AUROC] for binary classification). The prediction tasks used to assess LLMs were based on datasets from multiple biomedical domains, each representing distinct analytical challenges (Figure 1; Table S3). The first dataset (Q1) involved transcriptomics data obtained from the Gene Expression Omnibus (GEO), requiring parsing and preprocessing before downstream analysis. The second dataset (Q2) focused on epigenetics, specifically methylation data for the prediction of placental gestational age using regression models. The third dataset (Q3) was used for two classification tasks based on relative microbial abundance. Each of these prediction tasks tested the ability of LLMs to retrieve and organize data, foresee the need for data conversion, identify a suitable modeling framework, and select the appropriate package for fitting those modeling strategies across different programming environments.

Model evaluation

The test set accuracy metrics obtained by executing the code generated by LLMs for all tasks and programming languages are shown in Figure 2 and Table S4. Comparisons of predicted vs. actual values for Q1 and Q2 and true-positive vs. false-positive rates for Q3 are shown in Figure 3. The empty cells in

CellPress
OPEN ACCESS

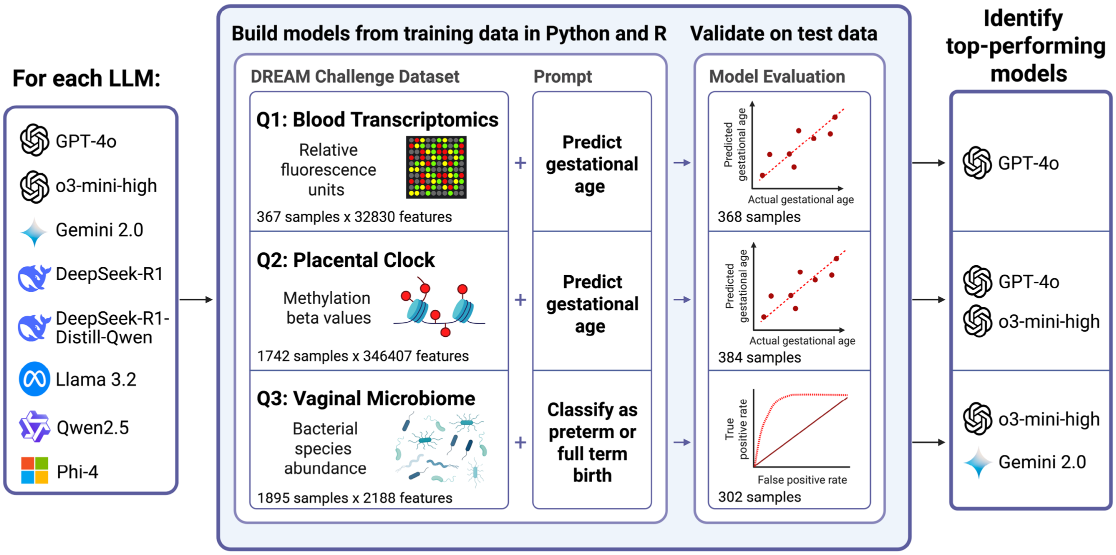

Figure 1. Study flow chart

Eight LLMs were prompted to generate R and Python code to fit and evaluate predictive models for four tasks. Q1: predict gestational age from blood transcriptomics. Q2: predict placental age from DNA methylation data. Predict (Q3A) preterm birth or (Q3B) early preterm birth from microbial relative abundance data. Analysis code was executed, and LLMs were ranked by code functionality and model accuracy. Created with BioRender. See also and Tables S1–S3.

Figures 2A and 2B signify that the code generated by the given LLM resulted in an error, preventing the completion of the task. Reasons for failures during the execution of LLM-generated code included attempts to load non-existent packages for R/python, inability to download data from the GEO repository or extract required metadata (specifically for python as Bioconductor/R package vignettes provide code examples for GEO data loading and parsing), attempts to select variables that did not exist in parsed datasets, failure to merge feature data with sample metadata before model fitting, and errors in the function calls for model fitting or plotting of results. Overall, among the four LLMs that generated code that successfully completed any task (o3-mini-high, 4o, DeepSeekR1, and Gemini), the R code generated by the LLMs was more successful in completing the four tasks (14/16) compared to Python (7/16). In three instances, LLMs created R code that successfully generated models and test set predictions but failed to produce code to plot results. Due to errors in retrieving gene expression data or parsing its corresponding metadata from GEO (Q1) in Python, none of the LLMs were able to complete this task. However, due to the efficient implementations of high-dimensional models (such as Ridge Regression), the RMSE in Python for task Q2 obtained by 4o and DeepSeekR1 was lower (i.e., higher accuracy) than that of the best-performing team in the Placental Clock DREAM challenge (Q2 RMSE: LLM top 1.12 vs. participant median 2.5 and top participant 1.24 weeks). These results align with previous studies highlighting the effectiveness of high-dimensional regression models in biomedical applications.[23] The top LLM prediction metrics for the other three tasks were comparable to or better than those of the median performance across DREAM challenge participants, but worse than those of the top teams (Q1 RMSE, LLM top = 5.42 vs. participant median 5.4 and top 4.5; Q3A AUROC, LLM top = 0.57 vs. participant median 0.58 and top 0.68; Q3B AUROC, LLM top = 0.59 vs. participant median 0.58 and top 0.92) (Figure 2; Table S4). Top participant models were significantly more accurate than top LLM models for Q1 ($p < 0.001$) and Q3B ($p = 0.008$) but not Q3A ($p = 0.08$), while the top LLM model was more accurate than top participant model for Q2 ($p = 0.02$).

The ability of LLMs to generate R/python code that could be run successfully, resulted in accurate test predictions, and generated a visualization of the predictions was the basis for scoring and ranking of the LLMs (STAR Methods; Table 1). Out of all the LLMs we tested, o3-mini-high received the highest overall score (33), followed by 4o (23), DeepSeekR1 (22), and Gemini2.0 FlashThinking (19) (Table 1). Of note, obtaining code from successful LLMs typically took between a few seconds and 2 min, which is orders of magnitude faster than human participants could develop comparable code and significantly shorter than the 3 months allotted for submissions to the DREAM challenges. In general, LLM generated code was about one page per task and was easy to follow as it often included comments before each code chunk. The most challenging task in terms of data size (Q2, ∼360,000 methylation features and ∼2,000 samples for training) was handled particularly efficiently by the most successful LLMs (DeepseekR1 and 4o), both implementing a different version of ridge regression. The LLM code execution took a few hours rather than dozens of hours needed for fitting models by the top three teams in the Placental Clock DREAM challenge. The impact of the stochastic nature of analysis code generation by the top-performing LLM (o3-mini, high reasoning mode) was assessed by prompting three additional times the model with the same prompt. The success rate of completing the analysis tasks was identical (87.5%) to the one reported in the primary analysis (Figures 2A and 2B; Table S5), yet fluctuations in accuracy were noted as different types of

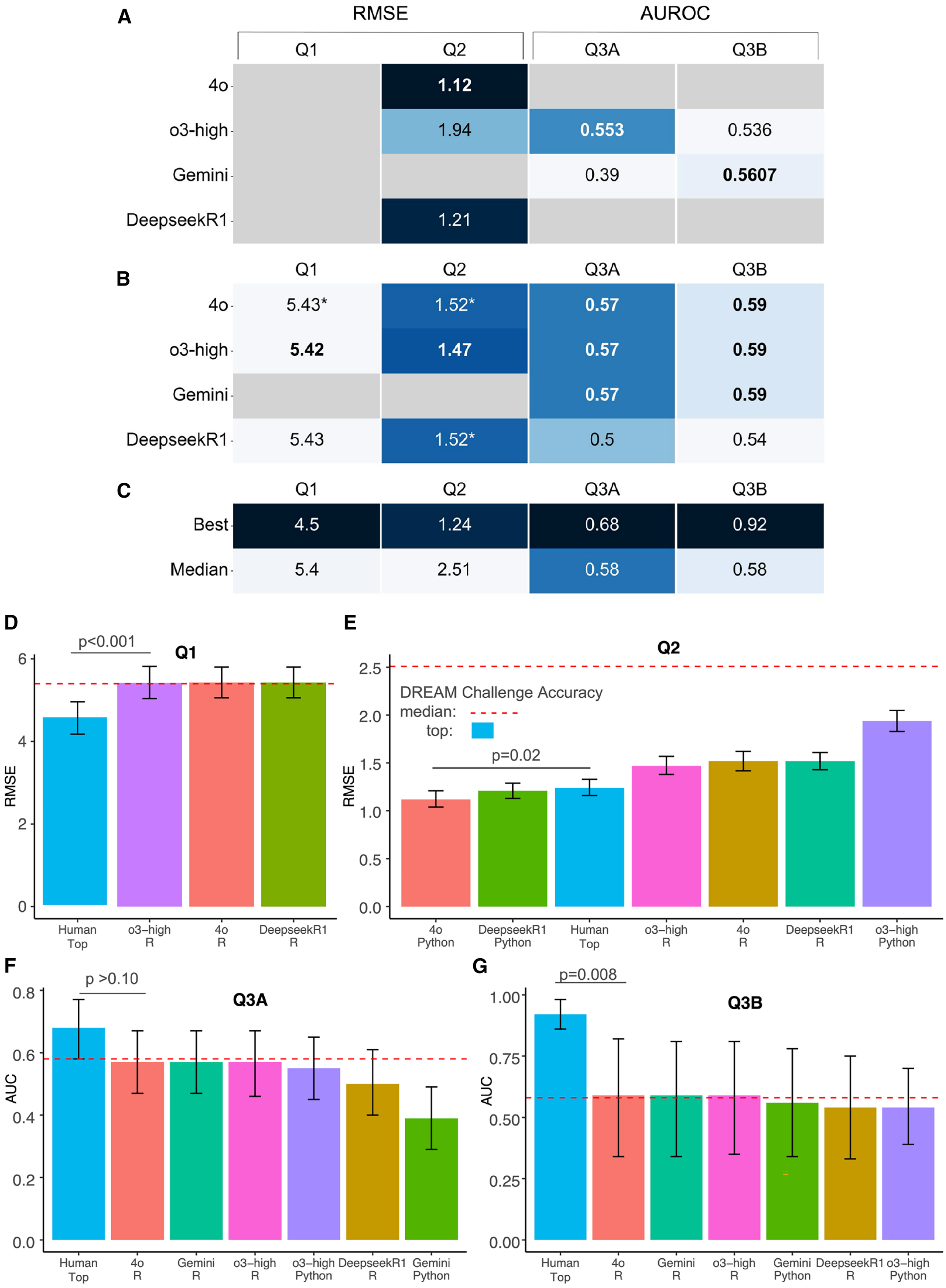

Figure 2. Test set prediction results

(A and B) RMSE and AUROC metrics were generated by executing LLM-generated Python (A) and R (B) analysis code. Colors are normalized by dataset, best scores are indicated by the darkest color per column, and the best score per dataset and language is shown in bold. Gray cells indicate that the code generated an error, preventing the completion of the task. Asterisks indicate that the model did not successfully generate a plot of the results. Models listed in Table S1 but not included here did not generate any successful code.

(legend continued on next page)

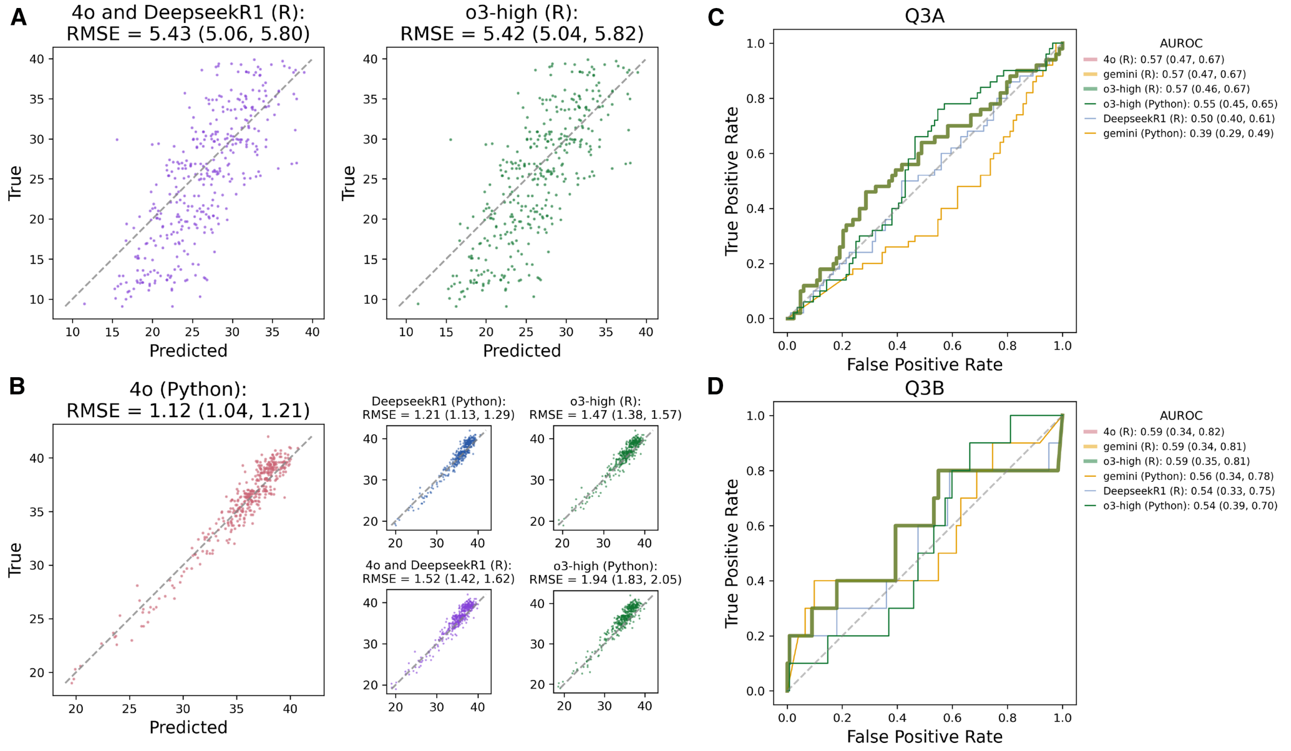

Figure 3. ROC curves and prediction scatterplots

(A) Predicted vs. actual gestational ages for all successful LLMs for Q1.

(B) Predicted vs. actual placental gestational ages for the top-scoring LLM for Q2 (left) and lower-scoring LLMs (right subplots). 4o- and DeepseekR1-generated code output identical predictions for (A) and (B) and are plotted together in purple.

(C) Receiver operating characteristic (ROC) curves for all LLM-generated predictions for Q3A endpoint.

(D) ROC curves for all LLM-generated predictions for Q3B endpoint.

In (C) and (D), the three highest scoring models output identical predictions and are represented by the bold green line. Confidence intervals for RMSE statistics were obtained using bootstrap (1,000 iterations), while for the area under the ROC (AUROC), they were obtained using the DeLong method. See also Table S4.

predictive models or tuning techniques were used. The accuracy reported in the initial analysis for the 7 completed tasks in Figures 2A and 2B matched the average accuracy over the 3 trials in 5/7 tasks, exceeded it in 1/7, and underestimated it in 1/7 tasks.

DISCUSSION

Our benchmarking study leveraging omics data from DREAM challenges highlights both the promise and limitations of LLMs in biomedical machine learning applications. Similar benchmarking approaches have been employed to assess LLM performance in other domains, including chemistry and clinical medicine.[24,25] Herein, we found that API-based LLMs such as o3-mini-high and 4o outperformed smaller locally run models in execution success and accuracy, reinforcing the advantages of larger cloud-hosted architectures. However, local models offer increased control and customization, which may be advantageous for researchers seeking data privacy or fine-tuned execution. Prior research has shown similar trade-offs in LLM performance across different computing environments.[26]

In terms of test set prediction accuracy of models obtained with LLM-generated analysis code compared to human-generated code, our results suggest that in some instances, LLM-generated models can be more accurate and faster to develop. For task Q2, where the LLM-generated model from ∼350,000 methylation features was more accurate than the human counterpart (RMSE = 1.12 vs. 1.24, respectively), neither the LLM nor the human had access to the test set or had any feedback on their model accuracy based on the test set. For this task, the best human-developed model was based on multi-stage random forest models, each of them utilizing thousands of methylation features, and leveraging additional clinical information, such as the obstetrical complication involved in the

(C) The best and the median model accuracy achieved by teams in the corresponding DREAM challenges.

(D–G) Bar plot representations with 95% confidence intervals of the same performance metrics in (A)–(C) except that median DREAM challenge participant metrics are shown as red dotted lines instead of a bar. *p* values indicate the significance of differences between performance metrics of the top participant in the DREAM challenges and the top LLM for each task. Underlying data are presented in Table S4, and results for three additional technical replicates for o3-mini-high are included in Table S5.

Table 1. LLM scoring

	Python code				R code				
Model	Q1 a|b|c|d	Q2 a|b|c|d	Q3A a|b|c|d	Q3B a|b|c|d	Q1 a|b|c|d	Q2 a|b|c|d	Q3A a|b|c|d	Q3B a|b|c|d	Model Score
o3-mini-high	0	1|2|0|1	1|2|1|1	1|2|0|1	1|2|1|1	1|2|1|1	1|2|1|1	1|2|1|1	33
4o	0	1|2|1|1	0	0	1|2|1|0	1|2|1|0	1|2|1|1	1|2|1|1	23
DeepSeekR1	0	1|2|1|1	0	0	1|2|1|1	1|2|1|0	1|2|0|1	1|2|0|1	22
Gemini	0	0	1|2|0|1	1|2|1|1	0	0	1|2|1|1	1|2|1|1	19
DeepSeekDistill	0	0	0	0	0	0	0	0	0
Llama	0	0	0	0	0	0	0	0	0
Phi-4	0	0	0	0	0	0	0	0	0
Qwen	0	0	0	0	0	0	0	0	0

For each task (Q1, Q2, Q3A, Q3B), models were evaluated for their ability to produce Python and R code that successfully (1) extracted and formatted data (1 point); (2) trained a model, applied it to the test set, and calculated the performance metric (2 points); (3) generated the required plot (1 point); and (4) achieved an accuracy that was within one significant digit of the best model for the endpoint (1 point). The maximum score for a+b+c+d was 5 points (1+2+1+1).

pregnancy. Such clinical information was not provided to the challenge participants but was publicly available for the training data. In contrast, the best LLM (OpenAI's 4o) used a Ridge Regression model including only the placenta methylation features. Moreover, LLMs were neither prompted to use the additional clinical information nor made aware of their availability.

For the other three prediction or classification tasks, the human participants in the DREAM challenges had the advantage of either using additional demographics, feature data, and time points in the models (Q3A and B) or receiving and using feedback on their model accuracy based on the test set accuracy (Q1 and Q3A and B). Typically, participants in DREAM challenges can select as their final submission the model that achieved the highest performance among multiple trials, with 3–5 trials typically allowed. LLM performance was assessed in a single trial.

One of the key motivations for integrating LLMs into the biomedical research process is their ability to support computational tasks by allowing prototyping of analysis code for researchers without coding skills and improving the productivity of those with coding skills. While crowdsourcing has democratized access to datasets and allows assessment of models on blinded data while facilitating global collaboration, it also introduces challenges related to reproducibility, coding variability, and quality control.[27] LLMs offer a potential solution by generating standardized, executable code, reducing the variability inherent in manually coded solutions, although some level of variability is inherent also to LLM generation due to their stochastic nature. Furthermore, LLMs can automate complex preprocessing steps, ensuring consistency in data handling across multiple modeling approaches, a known limitation of crowdsourced competitions.[28]

This is not to say that usage of LLMs does not come with its own limitations. For instance, while this study utilized various types of molecular and lab-test data, these data were all in a tabular format and derived in relation to reproductive health. Thus, it is very possible that these same LLMs, when applied to other data types or conditions, may not perform as well. Similarly, this study prompted LLMs to build the predictive models in very specific ways, and it is likely that LLM-created workflows to build more complex models will not do as well. Since LLM outputs are stochastic by nature, a single generation (i.e., one shot, as we did in our study) is insufficient to reliably capture a model's actual capacity for code generation. In addition, an important caveat is that LLMs—particularly when trained or prompted in similar ways—may converge on a narrow set of model architectures or analytic approaches. Such convergence could inadvertently reinforce suboptimal solutions rather than encourage methodological diversity. The range of scoring we observed across models in this study suggests that, while there is still some variability in how LLMs approach the task, there is a nontrivial risk that future applications could favor reproducibility of "standardized" code at the expense of innovation or optimal task-specific performance. Explicitly recognizing this trade-off is essential as the field considers how best to integrate LLM-generated workflows into biomedical research.

This was illustrated in this study for task Q1 where 3 models achieved the same accuracy (RMSE, 5.4 weeks). Multiple identical models will lead to ties in the ranking of the participating teams and less diversity in the approaches and ultimately less insight into the question formulated in the DREAM challenge. However, the use of LLMs by scientists in their daily analysis tasks has the potential to streamline model development and avoid over-stating the accuracy of models, particularly in reproductive health research, where accurate and reproducible predictive models can significantly impact clinical decision-making and reliable biomarkers derived from omics studies are scarce. A common source for over-stating the accuracy is the feature selection bias, where most predictive features are selected using the full dataset and only then data are split into training and testing.[29] None of the four successful LLMs that generated code attempted to leak information between training and test sets. Future research should explore how LLMs can be further fine-tuned for other biomedical applications and using other data types. Additionally, the integration of LLM-generated models into clinical pipelines should be rigorously evaluated to assess their translational potential and ensure alignment with medical best practices. In parallel, there is a clear rationale for modeling with omics features rather than relying only on routinely collected clinical variables. Large, prospective cohorts show that high-dimensional proteomic or epigenomic signatures capture

subclinical biology and can improve discrimination and reclassification for future disease beyond age, vitals, anthropometrics, and standard labs. For example, sparse plasma-protein panels trained in the UK Biobank predicted risk across multiple common diseases and, in several endpoints, outperformed (or added to) models built from conventional clinical factors.[30]

As a result, combining routinely available clinical data with omics can yield the most useful—and clinically interpretable—models. A well-known example is RSClin in early breast cancer, which integrates a 21-gene expression score with clinical-pathologic variables to provide more individualized recurrence and treatment-benefit estimates than either source alone; analogous clinico-omics integrations are increasingly standard across disease areas.[31]

Finally, LLMs can help leverage routinely collected data by unlocking predictive signal in unstructured notes and imaging. Health-system-scale language models trained on electronic health record text (e.g., NYUTron) have improved multiple prospective predictions relative to strong baselines, while vision foundation models (e.g., RETFound) demonstrate label-efficient transfer to downstream retinal disease tasks—both illustrating how foundation/LLM approaches can enhance routine clinical data and facilitate integration with omics when appropriate.[32]

This study provides a comprehensive benchmarking of LLMs for biomedical predictive modeling, evaluating their ability to generate machine learning code across multiple data modalities and coding languages. By addressing key challenges associated with code and models generated from crowdsourcing in biomedical research, LLMs offer a promising avenue for improving reproducibility, standardization, and coding efficiency, yet they may lead to identical models generated by challenge participants who adopt LLMs. While API-based LLMs demonstrated superior reliability, locally run models provide opportunities for enhanced control. LLMs offer significant potential in automating machine learning workflows but require careful validation to ensure reproducibility and accuracy in biomedical research. As a result, while more advanced models emerge, capable of handling various kinds of data, continued oversight and validation of these models' ability to accurately analyze and interpret such data is essential. Ongoing advancements in LLM architectures and prompt engineering strategies are poised to further refine their utility in scientific computing, including efforts to address major public health challenges such as preterm birth.

Our work demonstrates that LLMs can be integrated into researchers' predictive modeling workflows by rapidly generating code when provided with precise prompts about data structure, variables, and outcomes. Advantages include faster development and model performance comparable to the participant median across coding languages. Challenges include the cost of advanced LLMs and data security and privacy. Future work should explore agentic AI, as opposed to single-shot LLM prompting evaluated herein. Agentic AI can iteratively refine models but will require careful consideration of secure data access and resource management.

Limitations of the study

Several limitations of this study should be noted. Despite assessing eight different LLMs, the range of options in this area is wide and we only covered a subset of the options available to users. Furthermore, although we covered three data domains, other predictive modeling scenarios could be considered within the genomics field (e.g., SNP data, proteomics). The predictive tasks were limited to the cross-sectional study design even when the original data were longitudinal (i.e., task Q3), and hence there is a need to further evaluate the ability of LLMs to handle more complex tasks such as multiple observations per subject, missing data, and multi-class outcomes. The need to keep the analysis task reasonably simple may have also put LLMs in a disadvantage for task Q3 where human counterparts used additional microbiome features and multiple samples per patient. Finally, the reproducibility analysis was limited to the top LLM (o3-mini); it is possible that code generation for the locally run, smaller models could have led to successful task completions if multiple runs were attempted with different temperature settings and seeds.

RESOURCE AVAILABILITY

Lead contact

Requests for further information and resources should be directed to and will be fulfilled by the lead contact, Marina Sirota (marina.sirota@ucsf.edu).

Materials availability

This study did not generate new, unique reagents.

Data and code availability

- This paper analyzes existing datasets; accession information for these datasets is listed in Table S2.
- All code is available at https://github.com/vtarca7/LLMDream (https://doi.org/10.5281/zenodo.18134749) and is publicly available as of the date of publication. This includes the code generated by all eight LLMs considered for each of the three DREAM challenges and two programming languages (48 files). Three replication trials of code generation for o3-mini-high for all endpoints and coding languages are also included. Output files including eventual errors and comments displayed by the R and Python interpreters when executing LLM code are also available in the same repository. Original figures generated by LLMs, already summarized in Figure 3, are included also in this repository.
- Any additional information required to reanalyze the data reported in this paper is available from the lead contact upon request.

ACKNOWLEDGMENTS

This work was funded by the March of Dimes Prematurity Research Center at UCSF and by ImmPort. Research reported in this publication was also supported by the Eunice Kennedy Shriver National Institute Of Child Health & Human Development of the National Institutes of Health under award number R21HD115800 (A.L.T.). The contributions of the NIH author (R.R.) were made as part of his official duties as NIH federal employee, are in compliance with agency policy requirements, and are considered works of the US government. However, the findings and conclusions presented in this paper are those of the authors and do not necessarily reflect the views of the NIH or the US Department of Health and Human Services.

AUTHOR CONTRIBUTIONS

Conceptualization, M.S., T.T.O., and A.L.T.; methodology, A.L.T., G.B., G.S., M.S., and T.T.O.; investigation, R.S., V.T., and C.A.D.; writing – original draft, R.S., C.A.D., N.K., V.T., A.L.T., T.T.O., and G.B.; writing – review & editing, C.A.D., M.S., T.T.O., A.L.T., R.R., and G.S.; funding acquisition, M.S.,

T.T.O., and A.L.T.; resources, M.S., T.T.O, R.R., and A.L.T.; supervision, M.S., S.B., A.B., A.L.T., and T.T.O.

DECLARATION OF INTERESTS

The authors declare no competing interests.

STAR★METHODS

Detailed methods are provided in the online version of this paper and include the following:

- KEY RESOURCES TABLE
- METHOD DETAILS
  - LLM prompting for R and python code generation
  - R and python code curation
- QUANTIFICATION AND STATISTICAL ANALYSIS
  - R and python code execution and LLM scoring
  - Statistical analysis
- ADDITIONAL RESOURCES

SUPPLEMENTAL INFORMATION

Supplemental information can be found online at https://doi.org/10.1016/j.xcrm.2026.102594.

Received: July 10, 2025
Revised: October 17, 2025
Accepted: January 9, 2026
Published: February 17, 2026

REFERENCES

1. Ohuma, E.O., Moller, A.-B., Bradley, E., Chakwera, S., Hussain-Alkhateeb, L., Lewin, A., Okwaraji, Y.B., Mahanani, W.R., Johansson, E.W., Lavin, T., et al. (2023). National, regional, and global estimates of preterm birth in 2020, with trends from 2010: a systematic analysis. Lancet *402*, 1261–1271. https://doi.org/10.1016/S0140-6736(23)00878-4.
2. Wang, R., Pan, W., Jin, L., Li, Y., Geng, Y., Gao, C., Chen, G., Wang, H., Ma, D., and Liao, S. (2019). Artificial intelligence in reproductive medicine. Reproduction *158*, R139–R154. https://doi.org/10.1530/REP-18-0523.
3. Curchoe, C.L., Flores-Saiffe Farias, A., Mendizabal-Ruiz, G., and Chavez-Badiola, A. (2020). Evaluating predictive models in reproductive medicine. Fertil. Steril. *114*, 921–926. https://doi.org/10.1016/j.fertnstert.2020.09.159.
4. Ngo, T.T.M., Moufarrej, M.N., Rasmussen, M.-L.H., Camunas-Soler, J., Pan, W., Okamoto, J., Neff, N.F., Liu, K., Wong, R.J., Downes, K., et al. (2018). Noninvasive blood tests for fetal development predict gestational age and preterm delivery. Science *360*, 1133–1136. https://doi.org/10.1126/science.aar3819.
5. Lee, Y., Choufani, S., Weksberg, R., Wilson, S.L., Yuan, V., Burt, A., Marsit, C., Lu, A.T., Ritz, B., Bohlin, J., et al. (2019). Placental epigenetic clocks: estimating gestational age using placental DNA methylation levels. Aging *11*, 4238–4253. https://doi.org/10.18632/aging.102049.
6. Papageorghiou, A.T., Kemp, B., Stones, W., Ohuma, E.O., Kennedy, S.H., Purwar, M., Salomon, L.J., Altman, D.G., Noble, J.A., Bertino, E., et al. (2016). Ultrasound-based gestational-age estimation in late pregnancy. Ultrasound Obstet. Gynecol. *48*, 719–726. https://doi.org/10.1002/uog.15894.
7. Honest, H., Hyde, C.J., and Khan, K.S. (2012). Prediction of spontaneous preterm birth: no good test for predicting a spontaneous preterm birth. Curr. Opin. Obstet. Gynecol. *24*, 422–433. https://doi.org/10.1097/GCO.0b013e328359823a.
8. Savitz, D.A., Terry, J.W., Dole, N., Thorp, J.M., Siega-Riz, A.M., and Herring, A.H. (2002). Comparison of pregnancy dating by last menstrual period, ultrasound scanning, and their combination. Am. J. Obstet. Gynecol. *187*, 1660–1666. https://doi.org/10.1067/mob.2002.127601.
9. Romero, R., Conde-Agudelo, A., Da Fonseca, E., O'Brien, J.M., Cetingoz, E., Creasy, G.W., Hassan, S.S., and Nicolaides, K.H. (2018). Vaginal progesterone for preventing preterm birth and adverse perinatal outcomes in singleton gestations with a short cervix: a meta-analysis of individual patient data. Am. J. Obstet. Gynecol. *218*, 161–180. https://doi.org/10.1016/j.ajog.2017.11.576.
10. Esplin, M.S., Elovitz, M.A., Iams, J.D., Parker, C.B., Wapner, R.J., Grobman, W.A., Simhan, H.N., Wing, D.A., Haas, D.M., Silver, R.M., et al. (2017). Predictive accuracy of serial transvaginal cervical lengths and quantitative vaginal fetal fibronectin levels for spontaneous preterm birth among nulliparous women. JAMA *317*, 1047–1056. https://doi.org/10.1001/jama.2017.1373.
11. Campbell, F., Salam, S., Sutton, A., Jayasooriya, S.M., Mitchell, C., Amabebe, E., Balen, J., Gillespie, B.M., Parris, K., Soma-Pillay, P., et al. (2022). Interventions for the prevention of spontaneous preterm birth: a scoping review of systematic reviews. BMJ Open *12*, e052576. https://doi.org/10.1136/bmjopen-2021-052576.
12. Tekola-Ayele, F., Biedrzycki, R.J., Habtewold, T.D., Wijesiriwardhana, P., Burt, A., Marsit, C.J., Ouidir, M., and Wapner, R. (2025). Sex-differentiated placental methylation and gene expression regulation has implications for neonatal traits and adult diseases. Nat. Commun. *16*, 4004. https://doi.org/10.1038/s41467-025-58128-3.
13. Meyer, P., and Saez-Rodriguez, J. (2021). Advances in systems biology modeling: 10 years of crowdsourcing DREAM challenges. Cell Syst. *12*, 636–653. https://doi.org/10.1016/j.cels.2021.05.015.
14. Tarca, A.L., Pataki, B.Á., Romero, R., Sirota, M., Guan, Y., Kutum, R., Gomez-Lopez, N., Done, B., Bhatti, G., Yu, T., et al. (2021). Crowdsourcing assessment of maternal blood multi-omics for predicting gestational age and preterm birth. Cell Rep. Med. *2*, 100323. https://doi.org/10.1016/j.xcrm.2021.100323.
15. Golob, J.L., Oskotsky, T.T., Tang, A.S., Roldan, A., Chung, V., Ha, C.W.Y., Wong, R.J., Flynn, K.J., Parraga-Leo, A., Wibrand, C., et al. (2024). Microbiome preterm birth DREAM challenge: Crowdsourcing machine learning approaches to advance preterm birth research. Cell Rep. Med. *5*, 101350. https://doi.org/10.1016/j.xcrm.2023.101350.
16. Bhatti, G., Sufriyana, H., Romero, R., Patel, T., Tekola-Ayele, F., Alsaggaf, I., Gomez-Lopez, N., Su, E.C.Y., Done, B., Hoffmann, S., et al. (2025). Placental epigenetic clocks derived from crowdsourcing: Implications for the study of accelerated aging in obstetrics. iScience *28*, 113181. https://doi.org/10.1016/j.isci.2025.113181.
17. Işık, E.B., Brazas, M.D., Schwartz, R., Gaeta, B., Palagi, P.M., van Gelder, C.W.G., Suravajhala, P., Singh, H., Morgan, S.L., Zahroh, H., et al. (2023). Grand challenges in bioinformatics education and training. Nat. Biotechnol. *41*, 1171–1174. https://doi.org/10.1038/s41587-023-01891-9.
18. Attwood, T.K., Blackford, S., Brazas, M.D., Davies, A., and Schneider, M.V. (2019). A global perspective on evolving bioinformatics and data science training needs. Brief. Bioinform. *20*, 398–404. https://doi.org/10.1093/bib/bbx100.
19. Lu, M.Y., Chen, B., Williamson, D.F.K., Chen, R.J., Ikamura, K., Gerber, G., Liang, I., Le, L.P., Ding, T., Parwani, A.V., et al. (2024). A foundational multimodal vision language AI assistant for human pathology. Nature *634*, 466–473. https://doi.org/10.1038/s41586-024-07618-3.
20. Tanno, R., Barrett, D.G.T., Sellergren, A., Ghaisas, S., Dathathri, S., See, A., Welbl, J., Lau, C., Tu, T., Azizi, S., et al. (2025). Collaboration between clinicians and vision–language models in radiology report generation. Nat. Med. *31*, 599–608. https://doi.org/10.1038/s41591-024-03302-1.
21. Singhal, K., Azizi, S., Tu, T., Mahdavi, S.S., Wei, J., Chung, H.W., Scales, N., Tanwani, A., Cole-Lewis, H., Pfohl, S., et al. (2023). Large language models encode clinical knowledge. Nature *620*, 172–180. https://doi.org/10.1038/s41586-023-06291-2.

22. Omiye, J.A., Gui, H., Rezaei, S.J., Zou, J., and Daneshjou, R. (2024). Large language models in medicine: the potentials and pitfalls: a narrative review. Ann. Intern. Med. *177*, 210–220. https://doi.org/10.7326/M23-2772.

23. Zhou, R.R., Wang, L., and Zhao, S.D. (2020). Estimation and inference for the indirect effect in high-dimensional linear mediation models. Biometrika *107*, 573–589. https://doi.org/10.1093/biomet/asaa016.

24. Chen, M., Tworek, J., Jun, H., Yuan, Q., Pinto, H.P. de O., Kaplan, J., Edwards, H., Burda, Y., Joseph, N., Brockman, G., et al. (2021). Evaluating large language models trained on code. Preprint at arXiv. https://doi.org/10.48550/arXiv.2107.03374.

25. Brown, T.B., Mann, B., Ryder, N., Subbiah, M., Kaplan, J., Dhariwal, P., Neelakantan, A., Shyam, P., Sastry, G., Askell, A., et al. (2020). Language models are few-shot learners. Preprint at arXiv. https://doi.org/10.48550/arXiv.2005.14165.

26. Bender, E.M., Gebru, T., McMillan-Major, A., and Shmitchell, S. (2021). On the dangers of stochastic parrots: can language models be too big? In Proceedings of the 2021 ACM Conference on Fairness, Accountability, and Transparency (ACM), pp. 610–623. https://doi.org/10.1145/3442188.3445922.

27. Wang, C., Han, L., Stein, G., Day, S., Bien-Gund, C., Mathews, A., Ong, J.J., Zhao, P.-Z., Wei, S.-F., Walker, J., et al. (2020). Crowdsourcing in health and medical research: a systematic review. Infect. Dis. Poverty *9*, 8. https://doi.org/10.1186/s40249-020-0622-9.

28. Barai, P., Leroy, G., Bisht, P., Rothman, J.M., Lee, S., Andrews, J., Rice, S.A., and Ahmed, A. (2024). Crowdsourcing with enhanced data quality assurance: an efficient approach to mitigate resource scarcity challenges in training large language models for healthcare. AMIA Jt. Summits Transl. Sci. Proc. *2024*, 75–84.

29. Ambroise, C., and McLachlan, G.J. (2002). Selection bias in gene extraction on the basis of microarray gene-expression data. Proc. Natl. Acad. Sci. USA *99*, 6562–6566. https://doi.org/10.1073/pnas.102102699.

30. Carrasco-Zanini, J., Pietzner, M., Davitte, J., Surendran, P., Croteau-Chonka, D.C., Robins, C., Torralbo, A., Tomlinson, C., Grünschläger, F., Fitzpatrick, N., et al. (2024). Proteomic signatures improve risk prediction for common and rare diseases. Nat. Med. *30*, 2489–2498. https://doi.org/10.1038/s41591-024-03142-z.

31. Sparano, J.A., Crager, M.R., Tang, G., Gray, R.J., Stemmer, S.M., and Shak, S. (2021). Development and validation of a tool integrating the 21-gene recurrence score and clinical-pathological features to individualize prognosis and prediction of chemotherapy benefit in early breast cancer. J. Clin. Oncol. *39*, 557–564. https://doi.org/10.1200/JCO.20.03007.

32. Jiang, L.Y., Liu, X.C., Nejatian, N.P., Nasir-Moin, M., Wang, D., Abidin, A., Eaton, K., Riina, H.A., Laufer, I., Punjabi, P., et al. (2023). Health system-scale language models are all-purpose prediction engines. Nature *619*, 357–362. https://doi.org/10.1038/s41586-023-06160-y.

33. R Core Team (2020). R: A Language and Environment for Statistical Computing (R Foundation for Statistical Computing) Version 4.4.2. .

34. Robin, X., Turck, N., Hainard, A., Tiberti, N., Lisacek, F., Sanchez, J.-C., and Müller, M. (2011). pROC: an open-source package for R and S+ to analyze and compare ROC curves. BMC Bioinf. *12*, 77. https://doi.org/10.1186/1471-2105-12-77.

STAR★METHODS

KEY RESOURCES TABLE

REAGENT or RESOURCE	SOURCE	IDENTIFIER
**Software and algorithms**		
R (v. 4.4.2)	R core team[33]	https://www.R-project.org/
Python (v. 3.11)	N/A	https://python.org/
pROC (v. 1.18.5)	Robin et al.[34]	https://cran.r-project.org/web/packages/pROC/index.html
LM Studio (v.0.3.16)	Element Labs Inc.	https://lmstudio.ai/
ChatGPT-4o	OpenAI	https://openai.com
ChatGPT-o3-mini (high reasoning)	OpenAI	https://openai.com
Deepseek-R1	DeepSeek	https://www.deepseek.com
Gemini 2.0 Flash (experimental thinking)	Google	https://gemini.google.com
Github repository for this study	This study	https://doi.org/10.5281/zenodo.18134749
**Other**		
DREAM Preterm Birth Prediction Challenge, Transcriptomics Dataset	Tarca et al.[14]	https://www.synapse.org/Syn1838082
Microbiome Preterm Birth DREAM Challenge Dataset	Golob et al.[15]	https://www.synapse.org/Syn26133770
Placental Clock DREAM Challenge Dataset	Bhatti et al.[16]	https://www.synapse.org/Syn59520082

METHOD DETAILS

LLM prompting for R and python code generation

LLMs were prompted using either a web-based API, or locally via LM Studio (v.0.3.16) on a Windows 11 Pro 64-bit system with 192 GB of RAM and 24 CPU cores (Table S1). All LLM prompts, whether executed locally or via APIs, were run using default temperature and seed settings.

R and python code curation

The LLM-generated code was saved and edited by a) adding extra lines of code to enable saving raw predictions (for successful LLMs only) and b) disabling any lines of code that attempted to install R or python packages. Saving raw predictions was done only for aesthetic purposes (Figure 3), whereas disabling code that attempted to install packages was needed to maintain consistency across the R and Python environments. Packages required by all LLMs were first installed before executing the analysis code. No other code edits were implemented to address possible sources of errors during the code execution, and the datasets did not have missing data that the LLMs would need to handle.

QUANTIFICATION AND STATISTICAL ANALYSIS

R and python code execution and LLM scoring

The code was executed in Python or R, using the same computing system (Red Hat Enterprise Linux 8.10 Ootpa, with 56 logical processors, 250 GiB of RAM) with all R/Python packages required by any LLM already installed. The test set accuracy and generated plots were then scored to enable LLM ranking. Two of the authors, with expertise in R (at the level of package contributor) and in Python, reviewed the outputs generated by the respective interpreters when executing the LLM-generated code, some of which did not run successfully. The scores were assigned as follows: (a) 1 point for successful data extraction and formatting, which included downloading the data from the repository, extracting the required metadata, identifying the relevant variables, and merging of feature data with the corresponding metadata), (b) 2 points for successful model training and application of the model on the test set and metric calculation, which involved training the model on the training dataset, testing it on the validation dataset, and calculating RMSE for regression tasks or AUROC for classification tasks, (c) 1 point for successful generation of the required plot, i.e., a graph illustrating the predictive performance of each model using RMSE (regression) or AUROC (classification), (d) 1 point for achieving highest prediction accuracy (within ±0.02 for AUROC and ±0.1 week for RMSE of the top model for that task).

Statistical analysis

Confidence intervals for test set RMSE statistics for tasks Q1 and Q2 were obtained using bootstrap (1,000 iterations), while for AUROC (Q3A, B) were obtained using DeLong method with the *pROC* package in R. Comparisons between top human participant model and top LLM model for Q1 and Q2 were performed using paired t-tests on absolute prediction errors, while for Q3A and Q3B using DeLong tests for paired ROC curves. Two-tailed tests were used in all instances with $p < 0.05$ used to infer significance.

ADDITIONAL RESOURCES

The LLM-generated code is available at https://github.com/vtarca7/LLMDream.

Cell Reports Medicine, Volume *7*

Supplemental information

Benchmarking large language models for predictive modeling in biomedical research with a focus on reproductive health

Reuben Sarwal, Victor Tarca, Claire A. Dubin, Nikolas Kalavros, Gaurav Bhatti, Sanchita Bhattacharya, Atul Butte, Roberto Romero, Gustavo Stolovitzky, Tomiko T. Oskotsky, Adi L. Tarca, and Marina Sirota

Supplementary Tables

Table S1: LLMs used in the comparison

LLM	Developer	Parameters	Run Method
Deepseek-R1	DeepSeek	671B	API
4o	OpenAI	Unknown	API
o3-mini-high	OpenAI	Unknown	API
Gemini2.0 FlashExpThink	Google	Unknown	API
Qwen2.5 Coder	Alibaba	14B	Local
Llama3.2	Meta	3B	Local
Phi-4	Microsoft	14B	Local
DeepSeek-R1-Distill-Qwen	DeepSeek	32B	Local

Table S1: Details the eight different LLMs tested for generating code for the 8 different prediction tasks. Developers of each LLM are listed, as well as the method by which the LLM was run (API vs. Local). When available, information on the number of parameters for each LLM is described as well. While GPT-4o is OpenAI's flagship multimodal model, designed for both high-level reasoning and seamless real-time multimodal interaction, o3-mini-high is a more specialized model for technical/logical reasoning within text—best used for accuracy on STEM, math, and programming. Sources: https://openai.com/index/openai-o3-mini/, https://openai.com/index/hello-gpt-4o/. **Related to** Figure 1 and STAR methods.

Table S2: Prompts and computational tasks

Task/Data type	Prompt to LLM
Q1:<br>Predict gestational age from gene expression in blood<br><br>~30k features	Write a program in **R/python** which downloads the Gene Expression Omnibus dataset GSE149440 and uses expression data of tens of thousands of genes in a few hundred samples to fit a prediction model of gestational age (gestational age:ch1) variable from the metadata. Only samples assigned to the training set (metadata variable train:ch1=='1') should be used for training. Apply the model on the test defined as metadata variable train:ch1==0. Print the root mean squared error (RMSE) on the test set and generate a scatter plot of the predicted vs actual gestational age values for the test set.
Q2<br><br>Predict gestational age from placenta methylation data<br><br>~360k features	Write **R/python** code that fits one predictive model for a continuous variable gestational age (GA) using about 400 thousand methylation features measured in about 2000 samples. The feature data for the training set is in df_training.csv while for the test set is in df_test.csv. Features are rows and samples are columns in the feature data, with the first column being the feature names. The metadata files are ano_training.csv and ano_test.csv. The feature data and metadata are linked by the Sample_ID. Do not use any other metadata variables in the models besides feature data. Print the root mean squared error (RMSE) on the test set and generate a scatter plot of the predicted vs actual gestational age values for the test set and include RMSE in the legend.
Q3A:<br>Predict Preterm Birth from microbial abundance<br><br>Q3B:<br>Predict Early Preterm Birth from microbial abundance<br><br>~2k features	Write **R/python** code that fits one predictive model for a binary outcome PTB using about two thousands microbiome features measured in a few hundreds of samples. The feature data for the training set is in training_species_abundance.csv while for the test set is in validation_species_abundance.csv. Discard features not in common between training and validation sets and do not use any other metadata in the models besides microbial feature data. The metadata for the training set is in training_metadata.csv while for the test set in validation_metadata.csv. The feature data and metadata are linked by the specimen column in these datasets. There are multiple observations (specimen) for each subject identified by the column participant_id. Do use only the entry with highest collect_wk value < 32 for each participant_id to fit and evaluate the models. PTB is defined as metadata variable delivery_wk < 37. Print AUC ROC value on the test set and plot an ROC curve. Repeat the analysis above to predict EarlyPTB outcome defined as delivery_wk < 32 using data for last specimen with collect_wk<28 for each participant_id.

Table S2: For each task, the main goal/endpoint is noted, along with the number of features in the three different datasets used. The exact prompt given to the LLMs is shown. **Related to** Figure 1 and STAR methods.

Table S3: Dataset information

Dataset	DREAM Challenge name	Accession Number	Number of samples		
			Train	Test	Total
**Q1**	Predict gestational age from blood transcriptomics data	Syn1838082<br>GSE149440	367	368	735
**Q2**	Predict gestational age from placental methylation data	Syn59520082	1742	384	2126
**Q3 (A and B)**	Classify term vs. preterm birth from vaginal microbiome data	Syn26133770<br>SDY2187<br>SDY465	1895	302	2197

Table S3: This table details the four different tasks each LLM was prompted to perform. Q1 task prompted LLMs to build a model to predict gestational age from transcriptomics data, Q2 prompted LLMs to predict gestational age again, but from methylation data, and Q3 prompted LLMs to build a model to classify preterm birth from microbiome data. The table also details the study accession numbers for each of the datasets, as well as the number of samples in the training, test, and full datasets. Syn is a synapse identifier (synapse.org); GSE is a Gene Expression Omnibus identifier. SDY is an ImmPort identifier (https://immport.org/). **Related to** Figure 1 and STAR Methods.

Table S4: Performance metrics for LLMs and top human models

Dataset	Predictor	RMSE/AUC	95% Confidence Interval
Q1	Human	**4.55**	(4.18, 4.96)
Q1	o3-high: R	5.42	(5.04, 5.82)
Q1	4o: R	5.43	(5.06, 5.80)
Q1	DeepseekR1: R	5.43	(5.06, 5.80)
Q2	4o: Python	**1.12**	(1.04, 1.21)
Q2	Human	1.24	(1.16, 1.33)
Q2	o3-high: R	1.47	(1.38, 1.57)
Q2	4o: R	1.52	(1.42, 1.62)
Q2	DeepseekR1: R	1.52	(1.43, 1.61)
Q2	o3-high: Python	1.94	(1.83, 2.05)
Q2	DeepseekR1: Python	1.21	(1.13, 1.29)
Q3A	Human	0.68	(0.58,0.77)
Q3A	4o: R	0.57	(0.47,0.67)
Q3A	Gemini: R	0.57	(0.47,0.67)
Q3A	o3-high: R	0.57	(0.46,0.67)

Q3A	o3-high: Python	0.55	(0.45,0.65)
Q3A	DeepseekR1: R	0.5	(0.40,0.61)
Q3A	Gemini: Python	0.39	(0.29,0.49)
Q3B	Human	**0.92**	(0.86,0.98)
Q3B	4o: R	0.59	(0.34,0.82)
Q3B	Gemini: R	0.59	(0.34,0.81)
Q3B	o3-high: R	0.59	(0.35,0.81)
Q3B	DeepseekR1: R	0.54	(0.33,0.75)
Q3B	Gemini: Python	0.56	(0.34,0.78)
Q3B	o3-high: Python	0.54	(0.39,0.70)

Table S4: Performance metrics with 95% confidence intervals for LMM models and top human models. Values in bold mean significantly better (more accurate) than the next ranked model. **Related to** Figure 2.

Table S5: Reproducibility of o3-mini-high LLM code generation

Task	Language	LLM run	Completed	Metric type	Value
1	R	Primary	1	RMSE	**5.42**
1	R	Repeat 1	1	RMSE	5.43
1	R	Repeat 2	1	RMSE	5.42
1	R	Repeat 3	1	RMSE	5.43
1	Python	Primary	0	RMSE	NA
1	Python	Repeat 1	0	RMSE	NA
1	Python	Repeat 2	0	RMSE	NA
1	Python	Repeat 3	0	RMSE	NA
2	R	Primary	1	RMSE	1.47*
2	R	Repeat 1	1	RMSE	1.51
2	R	Repeat 2	1	RMSE	1.51
2	R	Repeat 3	1	RMSE	1.26
2	Python	Primary	1	RMSE	**1.94**
2	Python	Repeat 1	1	RMSE	1.21
2	Python	Repeat 2	1	RMSE	2.52
2	Python	Repeat 3	1	RMSE	1.94

3A	R	Primary	1	AUC	**0.567**
3A	R	Repeat 1	1	AUC	0.50
3A	R	Repeat 2	1	AUC	0.56
3A	R	Repeat 3	1	AUC	0.56
3A	Python	Primary	1	AUC	0.55**
3A	Python	Repeat 1	1	AUC	0.394
3A	Python	Repeat 2	1	AUC	0.394
3A	Python	Repeat 3	1	AUC	0.394
3B	R	Primary	1	AUC	**0.587**
3B	R	Repeat 1	1	AUC	0.54
3B	R	Repeat 2	1	AUC	0.60
3B	R	Repeat 3	1	AUC	0.60
3B	Python	Primary	1	AUC	**0.54**
3B	Python	Repeat 1	1	AUC	0.54
3B	Python	Repeat 2	1	AUC	0.54
3B	Python	Repeat 3	1	AUC	0.54

Table S5: The completion of the analysis task (1: success; 0: failure) and performance metrics are shown for the primary analysis reported and for 3 additional repeats of analysis code generation. Bolded values of the primary analysis match the average of the 3 additional repeats. * indicates that RMSE in primary analysis was higher (lower accuracy) than the average of the 3 additional repeats. ** indicates that the

AUC value in the primary analysis was higher (better accuracy) than the average of the 3 repeats. **Related to** Figure 2.
